# 난임 여성의 간호 요구 측정 도구 개발 및 타당도 검정

**DOI:** 10.4069/kjwhn.2020.03.31.1

**Published:** 2020-06-02

**Authors:** Jummi Park, Nayeon Shin, Kyungmi Lee

**Affiliations:** 1 Department of Nursing, Namseoul University, Cheonan, Korea; 1 남서울대학교 간호학과; 2 Department of Nursing, CHA University Bundang CMH Medical Center, Seongnam, Korea; 2 차의과학대학교 분당차병원 간호국; 3 Department of Nursing, Samsung Medical Center, Seoul, Korea; 3 삼성서울병원 간호본부

**Keywords:** Infertility, Instrumentation, Needs assessment, Nursing, Validation study, 난임, 도구 개발, 요구도 조사, 간호, 타당화 연구

## Abstract

**Purpose:**

High-quality nursing care must be provided for women with infertility, and their nursing care needs must be identified. Although scales have been developed to assess infertility-related stress, quality of life, and psychosocial status, there is a lack of scales that assess the nursing care needs of women with infertility. The purpose of this study was to develop a needs assessment scale for nursing care in women with infertility and to verify its reliability and validity.

**Methods:**

The 250 subjects in this study were women with infertility recruited from four hospitals. The scale was developed following the framework of DeVellis, through a literature review, in-depth interviews, development of preliminary items, verification of content validity, development of secondary items, verification of construct validity, and extraction of the final items. Date were analyzed using item analysis, factor analysis, confirmatory factor analysis, Pearson correlation coefficients, and Cronbach’s alpha. Reliability was tested using Cronbach’s alpha, and validity was evaluated using item analysis, exploratory factor analysis, and criterion validity.

**Results:**

The final version of the nursing needs assessment scale for woman with infertility consisted of 18 items. Four factors (physical and psychological nursing needs, needs for information regarding treatment, needs for infertility-related understanding and concern, and supportive needs) explained 66.0% of the total variance. Cronbach’s alpha was .92 for the overall instrument and ranged from .88 to .91 for the subscales.

**Conclusion:**

These results suggest that this needs assessment scale for nursing care in women with infertility demonstrated acceptable validity and reliability and contained items suitable for assessing the level of nursing care needed by women with infertility.

## Introduction

### 연구 필요성

2018년 우리나라 합계 출산율(total fertility rate)은 0.98명으로, 2005년의 1.08명 이후 13년 만에 처음으로 1명 이하로 감소하여 경제협력개발기구(Organization for Economic Cooperation and Development, OECD) 회원국 중에서도 최하위의 저출산 국가에 속한다[1]. 선진국의 난임률과 비교해 보았을 때에도, 영국 8.6%, 독일 8.0%, 미국 6.7%에 비해 우리나라의 난임률은 13.2%로 1.5배 이상 높은 수준임을 알 수 있다[2].

난임 진단과 더불어 배란 유도, 배아 이식 등의 난임 시술 과정과 같은 치료 관련 검사 및 처치는 난임 여성의 신체적 고통 및 정서적 부담을 가중시킨다[3]. 또한 임신이 난임 치료의 최종 목표이므로[4] 성 만족도 감소 및 관계의 결여, 결혼 만족도의 감소 등을 초래하기도 한다. 이에, 다양한 난임 시술 관련 정보[5] 및 의료적 지원[6]과 같은 난임 관련 상담 및 간호가 필요한 실정이다.

난임 여성의 심리적 특성 평가에 사용되는 측정 도구로는 난임 스트레스 평가 도구[7], 삶의 질 평가 도구[8] 등이 있으나 난임 여성들의 간호 요구를 사정하기 위한 도구는 아직 개발되지 않은 상태이다. 간호 요구란 개인의 실제적인 건강 상태와 최적의 건강 수준 사이에 차이가 있을 때 최적의 상태라는 목표에 도달하기 위해 필요하다고 인식된 것이라 할 수 있다[9]. 난임 여성은 자신이 목표로 생각하는 건강 상태를 성취하기 위해 일반적으로 치료에 대한 정보적 요구, 증상 관리 요구, 심리 사회적 요구, 지지적 요구 및 영적 요구 등을 가지고 있다[10,11]. 특히 난임은 치료 과정에서 배아의 양상에 따라서 향후 치료 방향이 달라지기도 하므로 진단 초기와 시술 중에 대상자들의 간호 요구도가 달라질 수 있다[12,13]. 따라서 대상자들의 특성을 반영한 요구 중재 프로그램을 개발하고 난임 간호 시 이를 적용할 필요가 있다. 하지만 이러한 요구 중재 프로그램에 선행해야 할 것은 난임 여성이 필요로 하는 간호, 정보, 교육 및 지지 체계에 대한 요구의 정확한 측정이다.

난임 여성의 요구에 관한 문헌 고찰 결과, 난임 여성 간호 요구에 대한 연구는 주로 질적 연구나 혼종 방법을 사용한 면담이었다[13,14]. 선행 연구에서는 난임 여성의 요구를 대상자가 가지고 있는 문제로 보았으며, 난임 여성의 포괄적 요구를 사정하기보다는 정보 요구, 지지 요구, 정서적 요구 등 특정 영역의 요구를 단편적으로 파악하려 하였다[15]. 또한 간호 요구란 개인이 살고 있는 사회의 문화에 의해 영향을 받는 것으로 알려져 있음에도 불구하고[9], 난임 여성 요구 측정 도구에 난임 환자가 생활하고 있는 사회의 문화적 요소가 반영되었는지가 불분명하였다. 따라서 본 연구는 기존 문제점들을 보완하고 대상자의 요구에 근거한 맞춤형 간호 중재와 간호 교육을 제공하기 위해 난임 여성의 간호 요구를 측정할 수 있는 도구를 개발하여 신뢰도와 타당도를 평가하고자 한다.

### 연구 목적

본 연구의 목적은 난임 여성의 간호 요구를 측정할 수 있는 도구를 개발하고, 개발된 도구의 타당도와 신뢰도를 검증하는 것이다.

## Methods

Ethics statement: This study was approved by the Institutional Review Board of Namseoul University (NO. NSUIRB-201901-004). Informed consent was obtained from the participants.

### 연구 설계

본 연구는 DeVellis [16]가 제시한 도구 개발 절차에 근거하여 난임 여성의 간호 요구를 측정하는 도구를 개발하고 타당도와 신뢰도를 검증하는 방법론적 연구이다.

### 연구 절차

**Figure. 1.**
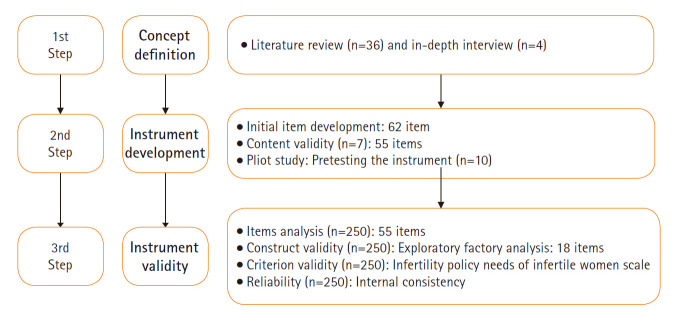
Flow of instrument development.

본 연구는 예비문항 작성을 위한 심층 면담과 예비조사, 본 조사의 3단계로 이루어졌다(Figure 1). 대상자의 구체적인 선정 기준은 난임 진단 후 난임 전문 병원에 내원하여 임신을 위하여 난임 치료를 받고 있는 만 20–50세 사이의 기혼 난임 여성으로 질문지의 내용을 이해하고 답할 수 있으며 본 연구의 목적과 방법 등에 대해 설명을 듣고 자발적으로 연구 참여에 서면 동의한 자이다. 정신적 질환을 진단받은 대상자는 자가 보고 응답으로 해당 사항을 확인한 후 대상자에서 제외하였다. 본 연구의 자료 수집이 이뤄진 4개 난임 전문 병원은 독립적인 IRB가 없어서 연구자가 속한 기관의 IRB의 심의 승인을 받고 4개 기관의 원내 조사 승인 하에 진행하였다. 원내 공고문을 게시하여 자발적으로 참여하는 대상자를 모집하였다. 연구자 3인이 외래 방문한 대상자에게 설명문을 제공하고 서면으로 동의서를 받은 후 설문지를 배부하였으며, 응답이 완료된 설문지는 밀봉 봉투에 넣어서 연구자들이 전달을 받았다. 설문조사가 끝난 후 본 연구에의 참여와 시간 할애에 대한 사례로 대상자들에게 1만원 상당의 선물을 제공하였다.

### 도구 개발 과정

#### 1차 예비문항 작성

난임 여성의 간호 요구를 연구한 국내∙외 문헌과 연구 논문을 고찰하였다. 데이터베이스는 Pubmed, Cumulative index to Nursing & Allied Health Literature (CINAHL), Google Scholar, Dbpia, Research Information Sharing Service (RISS), Korean Studies Information Service System (KISS) 등을 이용하였고 검색어는 ‘Needs assessment for infertility woman’, ‘needs scale for infertility women’, ‘난임 여성 간호 요구’로 하여 1990년도부터 2019년까지 검색된 문헌을 대상으로 하였다. 총 108편의 문헌이 검색되었고, 중복 문헌 32편을 제외한 76편을 선별하였다. 이후 초록 검토를 통하여 관련성이 적은 40편을 제외한 36편을 분석하였다(Supplement 1). 문헌 분석을 통하여 난임 여성에게 필요하다고 생각되는 간호 요구의 속성을 확인하였으며, ‘신체적 간호 요구’, ‘심리적 간호 요구’, ‘난임 치료와 관련된 정보 요구’를 도출하였다.

또한 난임 여성 간호 요구에 대한 문항을 도출하기 위해 난임 전문 병원에서 치료를 받고 있는 난임 여성 4명을 대상으로 1:1 심층 면담을 시행하였다. 면담은 난임 전문 병원의 병원장, 진료부장 및 간호부장에게 연구의 목적과 방법을 설명한 후 원내 조사 승인을 받고 연구자가 직접 시행하였다. 환자 대기실에 부착한 공고문을 통해 대상자가 면담에 대한 의사를 표시한 경우, 다음 진료 전 시간 약속을 하여 면담을 시행하였으며, 면담은 상담실에서 이루어졌다. 면담은 대상자별로 1, 2회 시행하였으며 평균 1시간 정도의 면담 시간이 소요되었다. 먼저, 반구조화된 질문지를 이용하여 면담 질문지를 작성하였다. 이는 ‘난임 상담 실태 조사 및 상담 서비스 제공을 위한 전달체계 마련 연구’ 보고서[10]를 토대로 작성하였으며, ‘신체적·심리적 간호 요구’, ‘난임 치료 정보 요구’, ‘난임 질환에 대한 교육 요구’, ‘지지적 요구’, ‘경제적 요구’를 중심으로 난임 여성의 간호 요구 영역을 사정하는 질문으로 구성하였다. 난임 여성에게 ‘난임 치료 시 어떤 부분이 제일 필요하고, 궁금하다고 생각하셨습니까?’라는 질문을 시작으로 심층 면담을 시행하였다. 심층 질문 구성 시 ‘난임 치료의 방법과 부작용에 관한 교육이 본인에게 얼마나 필요하다고 생각하십니까?’, ‘난임 치료 시 나타날 수 있는 증상에 관한 교육이 본인에게 얼마나 필요하다고 생각하십니까?’, ‘난임 치료를 받으면서 지지 체계가 본인에게 얼마나 필요하다고 생각하십니까?’ 등과 같은 질문들을 활용하여 난임 여성에게 필요한 간호 요구의 구성 요인을 개념화하였다. 이렇게 문헌 고찰과 심층 면담을 통해 도출된 자료에 기초하여 예비문항을 작성하였으며, 각 구성 요인별로 도출된 내용과 주제의 수에 비례하여 최소 10개에서 최대 20개까지 추출하여 총 74개 항목으로 구성하였다. 다음 단계로 간호대학 교수 1인과 실무 경력 20년 이상의 간호사 2인과 함께 중복된 문항을 제거하고 유사한 내용은 하나로 묶어 분류하는 작업을 통하여 5개 영역, 62개 문항으로 구성된 예비문항을 개발하였다. 이후 난임 여성에게 필요한 난임 간호 요구도의 구성 요인을 개념화하였다. 난임 간호 요구의 구성 요인은 ‘신체적·심리적 간호 요구’, ‘난임 치료 정보 요구’, ‘난임 질환에 대한 교육 요구’, ‘지지적 요구’, ‘경제적 요구’의 5가지 영역이었다.

#### 측정 범주 결정

본 도구는 Likert 4점 척도로 ‘전혀 필요하지 않다’ 1점부터 ‘매우 필요하다’ 4점까지로 응답하도록 하였다.

#### 내용 타당도 검증

전문가 집단 내용 타당도 검증을 위한 전문가의 수는 3–10명이 적합하다는 Lynn [17]의 연구에 근거하여 내용 타당도 검증을 위한 전문가 그룹을 구성하였다. 난임 여성을 돌보는 경력 20년 이상의 간호사 5명, 간호 대학 교수 2인으로 구성하였으며, 예비문항 62개 문항에 대해 각 문항의 내용 타당도 계수(Item-Content Validity Index, I-CVI)를 산출하여 분석하였다. I-CVI는 ‘적절하지 않음’ 1점, ‘관련성 적어 많은 수정이 필요함’ 2점, ‘관련성 있으나 약간의 수정이 필요함’ 3점, ‘매우 적절함’ 4점으로, 3점 또는 4점을 선택한 전문가의 수를 평가에 참여한 전문가의 총 수로 나누어 계산하였다. I-CVI .80 이상을 선정 기준으로 하였으며, 의미가 중복되거나 모호한 문항, 문항의 표현이 부적절한 7문항을 제외한 모든 문항이 I-CVI .80 이상이었다. 전문가 집단의 의견을 수렴하여 7개의 문항을 삭제한 후 55개 문항을 선정하였다.

#### 2차 예비문항 구성

예비조사 전, 선별된 문항에 대한 어휘의 정확성과 표현의 적절성을 판정하기 위해 국문학 전공자 1인의 자문을 통해 문법 및 어휘의 적절성, 단어의 띄어쓰기 등을 검토하였다. 예비조사는 난임 전문 병원에 내원한 난임 여성 10명을 대상으로 예비문항에 대한 이해 정도를 평가하였고, 설문에 소요되는 시간을 측정하였다. 설문 응답에 소요된 시간은 최소 5분에서 최대 15분이었고, 평균 8분이었다. 설문 문항에 대하여 전반적인 이해 정도는 5점 척도로 확인하였으며, ‘매우 이해하기 쉽다’, ‘이해하기 쉽다’, ‘보통이다’, ‘이해하기 어렵다’, ‘매우 이해하기 어렵다’ 중 이해하기 어렵다고 응답한 사람은 없었다.

### 평가 단계

내용 타당도와 예비조사를 통해 선별 후 수정된 문항으로 설문지를 구성하였다. 설문지는 대상자의 일반적 특성 및 난임 관련 특성 8문항과 본 연구에서 개발한 55개의 예비문항을 포함하여 총 63개의 문항으로 구성하였다. 설문 기간은 2019년 2월 1일부터 10월 31일까지로, 연구 대상자는 서울시에 있는 4개의 난임 치료 의료기관에 내원한 난임 여성 270명을 임의 표집하여 자료를 수집하였다. 이 중 25명의 설문지에서 일부 문항에 대한 응답 누락으로 250부의 자료를 최종 분석에 사용하였다. 구성타당도 검증을 위한 탐색적 요인 분석 수행 시 문항수의 최소 5배에서 최대 10배의 대상자가 필요하다[18]는 기준에 근거하였다.

### 자료 분석

개발된 측정 도구의 신뢰도와 타당도 검증을 위해 IBM SPSS Statistics for Windows, ver. 23.0 (IBM Corp., Armonk, NY, USA)을 이용하여 분석하였다. 대상자의 일반적 특성은 기술통계와 빈도 분석을 사용하였고, 탐색적 요인 분석은 각 요인별 평균과 표준편차를 확인하고 문항 간 상관관계(inter-item correlation)와 수정된 문항-총점 간 상관관계(corrected item-total correlation)를 확인한 후 수행하였다. 각 문항과 전체 문항 간의 상관계수가 .30 이상인 문항만을 선정하여 구성 타당도를 확인하였다. 선정 문항에 대한 탐색적 요인 분석 적합 여부는 Kaiser-Meyer-Olkim (KMO) 측도 값과 Bartlett 구형성 검증을 통해 판단하였다. 구성 타당도 검정을 위해 탐색적 요인 분석을 하였다. 추정 방법으로는 측정변수들이 다변량 정규분포를 이루었으므로 최대우도법을 적용하였다. 회전 방법은 직각회전 방식인 베리맥스(varimax) 방법을 사용하였다. 초기 문항 도출 시 간호 요구 영역을 5개로 구성하여 문항을 구성하였으므로 요인 수를 5개로 지정하여 산출하였다. 문항의 수렴 타당도와 변별 타당도 검정은 다특성-다방법 행렬을, 신뢰도는 Cronbach’s α 값을 이용하여 분석하였다.

## Results

### 일반적 특성

연구 대상자의 연령은 평균 36.4(±4.8)세로 최소 27세, 최대 46세였다. 세부적으로는 31세 이상 40세 이하의 연령군이 62.8%로 높았고, 학력은 대졸 및 이상이 대다수(96.0%)를 차지하는 고학력군이었다. 직업은 공무원, 전문직 등 출퇴근 직업이 있는 경우가 대다수(81.6%)였으며 대상자의 66.4%가 종교가 있었다. 임신 경험이 있는 대상자는 74.8%이었으며, 대상자의 62.0%는 유산 경험이 없었다. 난임 원인은 원인을 모르는 경우가 54.8%로 나타났다. 현재 치료 단계는 인공 수정(35.2%)이 가장 많았다(Table 1).

### 구성 타당도: 문항 분석

수집된 자료의 정규성을 확인하기 위해 각 문항의 왜도 및 첨도를 확인하였다. 분석 결과, 자료의 왜도값이 –1.31–0.71, 첨도값이 –.88–2.03으로, 왜도의 절대값이 2, 첨도의 절대값이 10을 넘지 않아 자료의 정규성 가정을 충족하였다[19].

다음으로, 문항의 기여도를 살펴보기 위해 문항 간 상관관계와 수정된 문항-총점 간 상관관계를 확인하였다. 55개 각 문항과 전체 문항 간 상관계수는 .33–.77의 분포를 보여 모든 문항의 상관계수가 .30 이상임을 확인하였고, 각 요인별 문항과 요인 간 상관계수도 .30 이상으로[19], 문항의 변별력을 보였다.

### 구성 타당도: 탐색적 요인 분석

55개 문항이 요인 분석을 하기에 적합한지 파악하기 위해 KMO의 표본적합성 값을 확인하고 Bartlett의 구형성 검정을 실시하였다. KMO값은 .93, Bartlett의 구형성 검정 결과 χ^2^=11,121.93 (*df*=1,485, *p*<.001)로 요인 분석을 하기에 적합하였다[20].

예비문항 작성 시 요인을 5개로 설정하여 문항을 개발하였으므로, 요인의 수를 5개로 설정하여 분석하였다. 문항 추출은 공통성 0.4 이상, 요인 부하량(factor loading) .40 이상, 요인 간 요인부하량이 .20보다 큰 차이를 기준으로 하였으며[21], 기준이 되는 결과 값은 회전된 요인 행렬로 하였다. 1차 요인 분석에서 26개 문항, 2차 요인 분석에서 7개의 문항이 기준에 적합하지 않아 삭제를 고려하였고, 해당 문항은 통계값 뿐만 아니라 뚜렷한 특성을 가지지 못한 문항이라는 연구자 판단 하에 삭제하였다. 3차 요인 분석에서 5개 요인, 22문항이 도출되었다. 이 중 1개 요인에 묶인 4개의 문항(‘난임과 한방’, ‘경제적 도움처 정보 제공’, ‘지방 거주자를 위한 편의 안내’, ‘대체요법의 종류’)이 하나의 요인으로 명명하기 어려웠고 여러 요인의 문항이 혼재되어 삭제하였다. 이에 최종 4개 요인, 18문항이 도출되었다. 요인 1은 고유값이 4.60이었고, 설명력은 24.2%였으며, 포함된 6문항들의 요인 적재값은 .63 이상이었다. 요인 2는 고유값이 3.20이었고 설명력은 16.8%였으며, 포함된 5문항들의 요인 부하량은 .63 이상이었다. 요인 3은 고유값 2.43, 설명력 12.7%이고, 4문항 요인 적재값은 .67 이상이었다. 요인 4는 고유값 2.33, 설명력 12.3%이고, 3문항 요인 적재값은 .73 이상이었다. 최종 4개 요인들의 누적 설명력은 66.0%로 나타났다(Table 2).

요인명은 개념화한 범주와 요인에서 가장 크게 적재된 문항을 참고로 명명하였다. 요인 1은 난임 치료와 관련된 신체적 증상, 건강 관리에 대한 간호 요구를 측정하는 문항으로 신체적 또는 심리적인 문제 해결에 대한 간호 요구를 포함하고 있어 ‘신체적·심리적 간호 요구’로 명명하였다. 총 6개의 문항으로 예비도구의 요인명과 일치하였으며 24.2%를 설명하고 있었다. 요인 2는 난임 검사 및 시술, 약물 투여 정보에 대한 간호 요구를 측정하는 문항으로 난임 치료에 대한 시술, 치료 과정에 대한 정보 습득에 대한 간호 요구를 포함하고 있어 ‘난임 치료 정보 요구’로 명명하였다. 총 5개 항목으로 예비도구의 요인명과 일치하였으며 16.8%의 설명 변량을 나타내고 있었다. 요인 3은 난임 질환에 대한 전반적인 이해와 관심사항에 대한 요구를 포함하고 있어 ‘난임 질환의 이해와 관심 사항에 대한 요구’로 명명하였다. 요인 3은 총 4개의 항목으로 설명 변량은 12.7%였다. ‘난임 질환에 대한 교육 요구’의 예비도구의 요인명과는 일치하지 않았다. 요인 4는 가족, 의료인, 친구 및 직장 동료의 지지체계에 대한 요구를 포함하고 있어 ‘지지적 요구’로 명명하였다. 요인 4는 총 3개의 항목으로 예비도구의 요인명과 일치하였으며 설명 변량은 12.3%였다.

### 수렴, 변별 타당도

다특성-다방법 행렬 결과 18개 문항과 이에 속한 요인과의 상관계수가 .75–.94의 값으로 모두 .40 이상으로 측정되어 문항 수렴 타당도를 확인하였다. 또한 각 문항이 자신이 속하지 않은 다른 하위 요인과의 상관계수는 .11–.61의 값들을 가지고 있는 것으로 측정되어 모든 문항이 각 문항이 속한 요인과의 상관계수보다 작은 값을 가지므로 변별 타당도가 확인되었다.

### 준거 타당도

본 연구에서 도구의 준거 타당도를 확인하기 위하여 개발된 난임 여성의 간호 요구 측정 도구와 이론적으로 유사한 불임 여성의 불임 정책 요구도 문항[22]을 저자의 사용 허락을 받은 후 상관 관계로 분석하였다. Pearson 상관계수는 .63 (*p*<.001)으로 의미 있는 상관관계를 보였으며, 4개의 하위 요인과 불임 여성의 불임 정책 요구도[22]와의 상관계수는 .44–.53으로 모두 중증도 수준이었다(Table 3).

### 신뢰도

측정 도구의 신뢰도 검증은 내적 일관성을 측정하는 Cronbach’s α 계수를 확인하였다. 최종 도구의 Cronbach’s α 계수는 .92였다. 각 요인별로 살펴보면 1요인 .91, 2요인 .88, 3요인 .89, 4요인 .89로 내적 일관성이 확보되었다[19] (Table 4).

### 최종 도구 확정

본 연구에서 도출된 난임 여성의 간호 요구 측정 도구는 4개의 하위 영역으로 이루어진 18개의 문항으로, 영역별 문항수가 다르므로 각 영역별 총점을 문항수로 나누어 영역별 평균 점수를 사용하며, 이를 통하여 난임 여성의 어느 영역의 간호 요구가 높은지 파악할 수 있다. 또한 영역별 평균 점수를 합산하여 총 점수가 높을수록 난임 간호에 대한 요구가 높다고 해석한다. ‘전혀 필요하지 않다’ 1점부터 ‘매우 필요하다’ 4점까지의 Likert 4점 척도로 측정하며, 영역별 평균 점수를 합산한 총점의 범위는 최소 18점에서 최대 72점으로 응답할 수 있도록 만들어졌다.

## Discussion

본 연구에서는 난임 여성의 간호 요구를 측정하기 위한 도구를 개발하여 내용 타당도, 안면 타당도, 구성 타당도 및 준거 타당도와 내적 일관성 신뢰도를 검증하였다. 연구 결과 타당성과 신뢰성의 근거를 확인할 수 있었으며, 최종적으로 4개의 하위 요인으로 구성된 18문항으로 난임 여성의 간호 요구를 측정할 수 있는 도구를 개발하였다.

개발된 도구의 탐색적 요인 분석을 통해서 구성 타당도를 검증한 결과, 누적 분산 비율은 66.0%였으며 하부 요인의 설명 분산 비율은 각각 24.2%, 16.8%, 12.7%, 12.3%였다. 탐색적 요인 분석 과정에서 삭제된 문항을 구체적으로 살펴보면, 19번 문항 “임신 반응 검사의 시기 및 방법”과 31번 문항 “난소 과자극 증후군 원인, 증상 및 치료법”, 25번 문항 “냉동 배아를 통한 임신 방법 및 절차”, 26번 문항 “냉동 배아 방법 및 임신 성공률”은 요인 적재값이 .40 미만이었다. 이들 문항은 구성 개념이 혼재되어 있었으며 각 하위 요인을 대표하지 못하는 문항으로 판단되어 삭제하였다. 또한 17번 문항 “난임 치료 후 부작용”은 신체적 간호 요구에 속하는 문항이었으나 같은 하부 요인의 12번 문항 “난임 치료제의 작용기전 및 효과, 부작용”과 13번 문항 “난임 시술의 종류 및 목적, 방법, 시술시간, 부작용”의 내용과 구성 개념이 중복되는 문항으로 판단하여 삭제하였다. 또한 41번 문항 “흡연”은 2개의 요인에 적재값이 비슷하게 측정되었으며, 공통성 값도 .27로 낮게 측정되어 삭제하였다.

탐색적 요인 분석 결과 확인된 4가지 요인 중 첫 번째 요인은 ‘신체적·심리적 간호 요구’로, 간호 요구의 전체 설명 분산의 24.2%로 가장 높은 설명력을 보였다. 적재된 문항은 난임 치료와 관련된 신체적 증상, 건강 관리에 대한 간호 요구를 측정하는 문항으로 ‘피로감, 수면장애’, ‘피부 변화(소양증, 두드러기)’, ‘복부 불편감(소화불량, 오심, 구토, 복부팽만, 복수)’, ‘불안, 우울, 자존감 저하’, ‘통증(생리통, 어깨 결림, 복통, 호흡곤란), ‘질 출혈’의 6문항으로 구성되었다. 제 1요인의 문항들은 난임 대상자들이 난임 치료를 받는 과정에서 가장 먼저 직면하게 되는 신체적 증상과 난임 치료 중에 느끼게 되는 심리적 문제를 경험할 때 요구되는 내용들이다. 난임 여성들이 치료 과정 중에 나타나는 신체적 증상 및 심리적 문제들을 어떻게 대처해 나가고 관리했느냐에 따라 임신 결과에도 영향을 미칠 수 있다는 점에서[21-23], 제 1요인은 난임 여성의 간호 요구에서 기본적으로 구성되어야 할 요인으로 탐색되었음을 알 수 있다.

두 번째 요인은 ‘난임 치료 정보 요구’로, 간호 요구의 전체 설명 변량 중 16.8%를 차지하여 두 번째로 높은 설명력을 보였다. 적재된 문항은 난임 검사 및 시술, 약물 투여 정보에 대한 간호 요구를 측정하는 문항으로 ‘난임 치료 약물의 작용 기전, 효과 및 부작용’, ‘남성 난임 검사’, ‘나팔관 촬영, 자궁경 검사, 복강경 검사와 같은 난임 진단 전 시술의 종류와 부작용’, ‘배란 유도제 투여 목적, 적응 대상자 및 방법’, ‘착상 호르몬 투여 목적, 적응 대상자 및 방법’, ‘호르몬 검사, 배란 검사와 같은 난임 진단 검사의 종류 및 방법’, ‘배란 유도, 인공수정, 시험관 시술과 같은 난임 진단 후 시술의 종류와 부작용’, ‘난임 치료 시작 시기’의 5가지 문항으로 구성되었다. 제 2요인의 각 문항들은 난임 여성이 의료기관에서 받는 인공수정, 체외수정, 보조생식 시술 및 각종 검사들의 유형과 배란 유도제, 착상 호르몬의 투여 목적 및 적응 대상자, 방법 등, 난임 치료 중인 여성들이 가장 정보를 받기를 원하는 요소로 가장 높은 설명력을 가진 요인으로 확인되었다. 특히 Park 등[24]의 간호사의 난임 간호에 대한 실무 교육 요구도 측정 도구 개발에서도 ‘난임 치료 및 증상에 대한 교육’이 난임 대상자들에게 실무 교육 요구도가 높은 요인으로 탐색되었는데, 이는 본 연구에서 ‘난임 치료 정보 요구’가 제 2요인으로 탐색되었음을 지지하는 결과라 할 수 있겠다.

세 번째 요인은 ‘난임 질환의 이해와 관심 사항에 대한 요구’로 간호 요구의 전체 설명 변량 중 12.7%를 차지하였다. 적재된 문항은 난임 질환의 이해와 관심 사항에 대한 요구를 측정하는 문항으로 ‘난임 치료 시작 시기’, ‘난임의 정의’, ‘난임의 원인’, ‘난임 자각 증상’, ‘난임 진단법’의 4가지 항목으로 구성되었다. ‘난임 질환에 대한 교육 요구’는 난임 상담 실태조사 및 상담 서비스 전달 체계 마련 연구 보고서에서도[10] 의료 정보 제공 상담 내용 중 난임의 원인, 진단과 같은 난임 질환에 대한 내용이 40.7%로 가장 높았는데 이는 난임 여성들이 난임에 대한 부정확한 정보들을 습득하지 않도록 의료진이 난임 전반에 대한 올바른 정보를 제공하여야 한다는 점을 시사한다.

네 번째 요인은 ‘지지적 요구’로 간호 요구의 전체 설명 변량 중 12.3%를 차지하였다. 적재된 문항은 난임 여성의 지지에 대한 간호 요구를 측정하는 문항으로 ‘가족의 지지’, ‘의료인의 지지’, ‘친구 및 직장 동료의 지지’의 3가지 문항으로 구성되었다. 선행 연구에서 난임 대상자들에게 가족의 지지는 회복 탄력성에 영향을 주고[25] 우울의 정도를 경감시키는 것으로 나타나[26] 난임 여성들에게 가족, 의료인 및 친구와 직장 동료의 지지 체계를 제공하고 이들의 지지적 간호 요구를 다차원적으로 파악하는 것이 중요하다는 것을 알 수 있다. 호주의 경우 난임 여성에게 정서적 지지를 제공하기보다는 지역사회 내 정신보건 의료 서비스 체제를 통해 난임과 관련된 정서적 고통에 대한 대처, 위기상황 발생 시의 해결책을 제시하는 데 중점을 두고 있다[10]. 또한 공적 의료 시스템 및 공적 보험이 발달한 유럽 국가들은 국고를 통해 심리적 상담을 포함한 일정 기간의 난임 지원을 하고 있으며, 호주 및 뉴질랜드의 경우에도 난임 여성들은 난임 시술 전 난임에 대한 전반적인 정보와 시술과 관련된 제반 사항에 대한 충분한 안내를 받고 치료적 정보 제공과 난임 치료 과정 결정 외에도 심리적 문제와 가족 지지에 관한 법제화된 도움을 받고 있다[27]. 반면 우리나라 난임 여성들의 경우, 국가의 지원이나 법제화된 도움을 통한 지지 체계를 제공받기 보다는 난임 치료 과정에 개입하는 의료진과의 상호작용에 의존하는 경우가 많다[24]. 특히 유교적 관념을 지닌 우리나라의 출산 문화와 관련하여 부모됨과 여성성의 문제[27], 부부 갈등, 사회적 관계와[10] 관련한 지지적 요구를 필요로 한다고 생각된다. 즉, 지지적 요구는 난임 환자가 생활하고 있는 사회의 제도적 환경과 문화적 특성에 따라 달리 해석될 수 있는데, 본 도구에서는 우리나라의 사회·문화적 특성을 고려한 가족의 지지, 의료인의 지지, 친구 및 직장 동료의 지지를 측정하는 문항이 포함되었고 이는 실제로 난임 치료를 받고 있는 난임 여성이 원하는 지지가 무엇인지 파악하는 데 실증적 도움을 줄 수 있는 항목이 될 수 있을 것이다. 제 4요인의 문항은 3개로 다른 하부 요인들의 문항수에 비해서 작지만, 4요인의 요인 부하값이 모두 .86 이상이며 회전 후에 모든 변인은 높은 요인 부하량을 갖는 측정 변수가 3개 또는 4개 이상 되는 것이 바람직하다는[2] 선행연구를 참고할 때 적합하다고 할 수 있겠다. 본 도구의 전체 18문항의 내적 일관성 신뢰도 Cronbach’s α는 .92로, 측정 문항 수가 적으면 신뢰도 계수가 낮아지는 경향이 있음에도 불구하고 신뢰도가 매우 좋은 것으로 나타났다. 각 하위 요인별 신뢰도도 .88–.92로 본 도구는 신뢰할 수 있는 도구임을 알 수 있다.

현재까지 난임 여성들을 대상으로 한 연구를 고찰해 보면 난임 스트레스, 불안, 우울 등 심리·정서적 측면을 측정하는 도구를 사용한 연구가 대부분으로, 난임 여성의 간호 요구를 측정한 도구는 전무하여 질적 연구 또는 혼종 방법을 사용하여 속성을 파악한 연구에 제한되었다. 또한 난임 대상자의 심리·정서적 측면을 측정하는 기존의 도구는[7,8] 난임 대상자의 실질적인 간호 요구도를 파악하거나 비교하는 데에도 제한점이 있었다. 반면에 본 연구에서 도출한 측정 도구는 도구개발 단계에 맞추어 개념을 정의하고 도구를 개발한 후 타당도와 신뢰도를 검증하였으므로, 추후 전 세계 가임 대상자의 10.0% 이상을 차지하고 있는[28] 난임 대상자의 간호 요구를 측정하는 데 적용성과 기여도 측면에서 큰 의의가 있다고 생각된다.

심리적 스트레스와 신체적 상실로 인해 삶의 질이 높지 않은 난임 여성은 치료 기간이 길어질수록 심리·정서적인 측면이 약해지고 치료 경과 과정에 대한 불안감이 높아지기 때문에 간호 요구에 대한 파악이 무엇보다 중요하다. 본 도구는 난임 여성이 요구하는 신체적 간호 요구, 사회·정서적 간호 요구, 교육적 요구, 치료적 요구를 다차원적으로 측정할 수 있으므로 추후 난임 여성의 간호 요구를 진단하거나 선별적으로 평가할 때 유용할 것으로 판단되며, 타당도와 신뢰도가 검증된 도구로 추후 간호 연구와 실무 분야에 도움이 될 것으로 기대한다.

본 연구의 제한점은 다음과 같다. 첫째, 도구 개발에서 타당도를 검증하는 방법으로 탐색적 요인 분석으로 요인의 구조를 확인한 후 새로운 대상자에게 다시 설문을 시행하여 확인적 요인 분석을 통해 요인 구조를 검증할 필요가 있으나, 본 연구에서는 탐색적 요인 분석을 할 수 있는 최소 자료를 수집한 상태로, 탐색적 요인 분석에서 도출된 문항으로 새로운 대상자에게 설문을 하지 못 하여 확인적 요인 분석은 이루어지지 않았다. 따라서 이를 보완할 수 있는 추가 연구를 제언한다. 둘째, 난임 전문 병원에 내원하지 않고 지역사회 내에서 보건소, 한방기관을 통한 치료를 하고 있는 난임 여성들을 포함하지 못하였고, 서울 및 수도권에 거주하는 난임 여성을 중심으로 표집하였기 때문에 연구 결과를 일반화하는 데 신중할 필요가 있다. 그러나 본 도구는 난임 여성의 신체적, 정서적 특성과 환경을 고려하여 개발한 도구이므로 난임 여성의 간호 요구를 정확히 파악하여 난임 여성을 위한 맞춤형 간호 중재 개발에 활용할 수 있다는 데 의의가 있다. 또한 본 도구는 난임 여성을 대상으로 측정하기 쉽고 간단한 문항으로 구성되어 있으므로 활용도와 유용성이 높다는 장점이 있다. 문항 수가 많으면 측정 오차가 높아지고 회수율이 낮아지는 경향이 있으므로, 본 도구는 난임 여성에게도 실무적으로 활용될 수 있는 가치가 있다고 판단된다. 난임 치료 전문 병원이나 지역사회의 사례 관리 프로그램에서 본 도구를 활용하여 난임 여성의 간호 요구를 조기에 선별하고 관리한다면, 이들의 난임 극복 및 임신 성공률 제고에 기여할 수 있을 것이다. 저출산 극복이 시급한 만큼 난임 여성의 간호 요구의 파악이 중요하게 여겨지는 현시점에서 난임 여성을 대상으로 간호 요구를 정할 수 있는 도구를 개발한 것은 시의적절하여 다양한 측면에서 기여할 수 있을 것으로 판단된다.

본 연구에서 개발한 난임 여성의 간호 요구 측정 도구는 다양한 방법을 통해 타당도와 신뢰도를 검증하였다. 본 도구는 총 18문항의 4개 하위 요인으로 구성된 4점 Likert식 도구로, 가능한 점수의 범위는 각 영역별로 1요인인 ‘난임 치료 정보 요구’는 6–24점, 2요인인 ‘신체적·심리적 간호 요구’는 5–20점, 3요인인 ‘난임 질환의 이해와 관심 사항에 대한 요구’는 4–16점, 4요인인 ‘지지적 요구’는 3–12점이며 점수가 높을수록 간호 요구도가 크다는 것을 의미한다. 본 도구는 신체적·심리적 증상, 난임 치료, 난임 질환, 지지적 체계에 걸쳐 다차원적으로 난임 여성의 간호 요구를 측정할 수 있으며, 각 영역별로 간호 요구가 무엇인지 파악할 수 있어 대상자별 특성에 맞는 간호를 제공하는 데에 기여할 수 있다. 최초로 난임 여성의 간호 요구를 측정할 수 있는 선별 도구로서의 활용 가능성이 높으므로 추후 난임 여성의 간호 요구에 대한 조기 파악과 관리에 사용할 수 있을 것이다. 더 나아가 난임 여성의 간호 요구와 관련된 중재를 개발하고 적용하여 그 효과를 평가할 때 유용하게 활용할 수 있으리라 기대한다. 간호 연구나 중재 프로그램에서 효과를 검증하기 위한 후속 연구를 제언하며, 본 도구는 국내 난임 여성들을 대상으로 수행한 연구이므로 추후 국외 난임 여성들에게 적용하고 측정하여 문화적 차이점을 파악할 것을 제언한다.

## Figures and Tables

**Table 1.** General characteristics of the participants (N=250)

Variable	Categories	n (%)
Age (year)	21–30	57 (22.8)
	31–40	157 (62.8)
	41 or older	36 (14.4)
Educational level	High school	10 (4.0)
	Undergraduate	198 (79.2)
	Graduate	42 (16.8)
Occupation	Housewife	46 (18.4)
	Full-time job	204 (81.6)
Religion	Yes	166 (66.4)
	No	84 (33.6)
Experience of pregnancy	Yes	187 (74.8)
	No	63 (25.2)
Experience of miscarriage/abortion	Yes	95 (38.0)
	No	155 (62.0)
Cause of infertility	Female factor	42 (16.8)
	Male factor	39 (15.6)
	Both female and male factors	32 12.8)
	Unknown	137 (54.8)
Type of infertility treatment	Ovulation induction	51 (20.4)
	Artificial insemination	88 (35.2)
	First in-vitro fertilization	54 (21.6)
	Repeated in-vitro fertilization	57 (22.8)

**Table 2.** Analysis of item appropriateness for the items and factors of the scale (N=250)

Factor	Item	Item and total correlation	Communality	Factor loading	Eigen value	Explained variance (%)
1. Physical and psychological nursing needs	35. Fatigue, sleep disorder	.69	.77	.83		
	36. Skin problems (pruritus, urticaria)	.69	.71	.79		
	37. Anxiety, depression, low self-esteem	.67	.69	.78		
	34. Abdominal discomfort (dyspepsia, nausea, vomiting, abdominal distension, ascites)	.62	.64	.75	4.60	24.2
	33. Pain (menstrual pain, stiff shoulder, abdominal pain, dyspnea)	.64	.59	.70		
	32. Vaginal bleeding	.60	.65	.63		
2. Needs for information on treatment	4. Symptoms of infertility	.67	.75	.77		
	5. Diagnosis of infertility	.62	.70	.74		
	2. Proper time to start infertility treatment	.65	.50	.73	3.20	16.8
	1. Definition of infertility	.59	.46	.63		
	3. Cause of infertility	.48	.69	.63		
3. Needs for infertility-related understanding and concern	13. Types and side effects of infertility treatment post-diagnosis (ovulation induction, artificial insemination, in-vitro fertilization)	.63	.75	.73		
	10. Types and side effects of infertility treatment pre-diagnosis (hysterography, laparoscopy)	.58	.71	.67		
	12. Mechanisms, effects, and side effects of infertility drugs	.72	.61	.67	2.43	12.7
	11. Purpose of administrating ovulation induction agents, patients for whom these agents are appropriate, and methods	.69	.92	.67		
4. Supportive needs	Support from family	.45	.76	.93		
	Support from healthcare providers	.49	.59	.82	2.33	12.3
	Support from friends and co-workers	.40	.57	.73		

**Table 3.** Criterion validity and reliability of the scale (N=250)

Nursing needs assessment scale for women with infertility	Policy needs for women with infertility scale	Cronbach's a
Total	.63	.92
Physical and psychological nursing needs	.53	.91
Needs for information on treatment	.51	.88
Needs for infertility-related understanding and concern	.48	.89
Supportive needs	.44	.89

**Table 4.** Correlations among subscales for the nursing needs assessment scale for women with infertility (N=250)

Factor	Item No.	Item and total correlation	Item and factor correlation			
			1	2	3	4
Physical and psychological nursing needs	35	.69	.89	.41	.54	.30
	36	.69	.85	.45	.48	.34
	37	.67	.84	.42	.46	.35
	34	.62	.83	.32	.46	.36
	33	.64	.83	.41	.46	.30
	32	.60	.77	.40	.45	.26
Needs for information on treatment	4	.67	.50	.87	.57	.18
	5	.62	.45	.84	.57	.11
	2	.65	.40	.85	.56	.27
	1	.59	.37	.79	.48	.29
	3	.48	.23	.75	.41	.25
Needs for infertility-related understanding and concern	13	.63	.47	.50	.87	.28
	10	.58	.38	.54	.84	.19
	12	.72	.57	.61	.89	.23
	11	.69	.56	.55	.88	.23
Supportive needs	44	.45	.36	.24	.20	.94
	45	.49	.39	.28	.24	.92
	46	.40	.29	.19	.28	.86
Cronbach's α			.92	.88	.89	.89

## Supplementary materials

Further details on supplementary materials are presented online (available at https://doi.org/10.4069/kjwhn.2020.03.31.1).

Supplement 1. List of selected literature (n=36).
