# Supplementary material for: Nursing needs assessment scale for women with infertility: development and validation
Source: Korean J Women Health Nurs. 2020 Jun 2;26(2):141–50. [Article in Korean] doi: 10.4069/kjwhn.2020.03.31.1 (PMC9328595; doi:10.4069/kjwhn.2020.03.31.1)
Supplement: Supplement 1. — List of selected literature (n=36). [file kjwhn-2020-03-31-1suppl1.pdf]

Supplement 1. List of selected literature (n=36)

S1. Peate M, Meiser B, Hickey M, Friedlander M. The fertility-related concerns, needs and preferences of younger women with breast cancer: a systematic review. Breast Cancer Res Treat. 2009;116(2):215-223. https://doi.org/10.1007/s10549-009-0401-6

S2. Benyamini Y, Gefen-Bardarian Y, Gozlan M, Tabiv G, Shiloh S, Kokia E. Coping specificity: the case of women coping with infertility treatments. Psychol Health. 2008;23(2):221-241. https://doi.org/10.1080/14768320601154706

S3. Jafarzadeh-Kenarsari F, Ghahiri A, Zargham-Boroujeni A, Habibi M. Exploration of the counseling needs of infertile couples: a qualitative study. Iran J Nurs Midwifery Res. 2015;20(5):552-559. https://doi.org/10.4103/1735-9066.164506

S4. Brucker PS, McKenry PC. Support from health care providers and the psychological adjustment of individuals experiencing infertility. J Obstet Gynecol Neonatal Nurs. 2004;33(5):597-603. https://doi.org/10.1177/0884217504268943

S5. Chang SN, Mu PF. Infertile couples' experience of family stress while women are hospitalized for ovarian hyperstimulation syndrome during infertility treatment. J Clin Nurs. 2008;17(4):531-538. https://doi.org/10.1111/j.1365-2702.2006.01801.x

S6. Bergart AM. The experience of women in unsuccessful infertility treatment: what do patients need when medical intervention fails? Soc Work Health Care. 2000;30(4):45-69. https://doi.org/10.1300/J010v30n04_04

S7. Jafarzadeh-Kenarsari F, Ghahiri A, Habibi M, Zargham-Boroujeni A. Exploration of infertile couples' support requirements: a qualitative study. Int J Fertil Steril. 2015;9(1):81-92. https://doi.org/10.22074/ijfs.2015.4212

S8. Burns LH. Psychiatric aspects of infertility and infertility treatments. Psychiatr Clin North Am. 2007;30(4):689-716. https://doi.org/10.1016/j.psc.2007.08.001

S9. Davis DC, Dearman CN. Coping strategies of infertile women. J Obstet Gynecol Neonatal Nurs. 1991;20(3):221-228. https://doi.org/10.1111/j.1552-6909.1991.tb02534.x

S10. Laffont I, Edelmann RJ. Perceived support and counselling needs in relation to in vitro fertilization. J Psychosom Obstet Gynaecol. 1994;15(4):183-188. https://doi.org/10.3109/01674829409025644

S11. Hynes GJ, Callan VJ, Terry DJ, Gallois C. The psychological well-being of infertile women after a failed IVF attempt: the effects of coping. Br J Med Psychol. 1992;65(Pt 3):269-278. https://doi.org/10.1111/j.2044-8341.1992.tb01707.x

S12. Daniluk JC. Strategies for counseling infertile couples. J Couns Dev. 1991;69(4):317-320. https://doi.org/10.1002/j.1556-6676.1991.tb01513.x

S13. Wischmann T. Implications of psychosocial support in infertility--a critical appraisal. J Psychosom Obstet Gynaecol. 2008;29(2):83-90. https://doi.org/10.1080/01674820701817870

S14. Mosalanejad L, Khodabakshi Koolee A. Looking at infertility treatment through the lens of the meaning of life: the effect of group logotherapy on psychological distress in infertile women. Int J Fertil Steril. 2013;6(4):224-231.

S15. Hasanpoor-Azghdy SB, Simbar M, Vedadhir A. The emotional-psychological consequences of infertility among infertile women seeking treatment: results of a qualitative study. Iran J Reprod Med. 2014;12(2):131-138.

S16. Korea University Research Management System. A study on the current status of infertility counseling and the development of infertility counseling delivery system. Cheongju: Ministry of Health and Welfare; 2016 Dec. Report No.: Q1609601.

S17. Lee YS, Kwon JH. A survey on the current status and demands of infertility counseling towards the development of an infertility counseling delivery system. J Korean Soc Matern Child Health. 2020;24(1):52-63. https://doi.org/10.21896/jksmch.2020.24.1.52

S18. Choi SM, Lee YS. The focus group interview on infertility experts for the development of infertility counseling system. J Korean Soc Matern Child Health. 2020;24(1):26-39. https://doi.org/10.21896/jksmch.2020.24.1.26

S19. Zargham-Boroujeni A, Jafarzadeh-Kenarsari F, Ghahiri A, Habibi M. Empowerment and sense of adequacy in infertile couples: a fundamental need in treatment process of infertility-a qualitative study. Qual Rep. 2014;19(6):1-14.

S20. Hirsch AM, Hirsch SM. The long-term psychosocial effects of infertility. J Obstet Gynecol Neonatal Nurs. 1995;24(6):517-522. https://doi.org/10.1111/j.1552-6909.1995.tb02389.x

S21. Romeiro J, Caldeira S, Brady V, Timmins F, Hall J. Spiritual aspects of living with infertility: a synthesis of qualitative studies. J

Clin Nurs. 2017;26(23-24):3917-3935. https://doi.org/10.1111/jocn.13813
S22. Hasanpoor-Azghady SB, Simbar M, Vedadhir AA, Azin SA, Amiri-Farahani L. The social construction of infertility among iranian infertile women: a qualitative study. J Reprod Infertil. 2019;20(3):178-190.
S23. Casu G, Ulivi G, Zaia V, Fernandes Martins MDC, Parente Barbosa C, Gremigni P. Spirituality, infertility-related stress, and quality of life in Brazilian infertile couples: analysis using the actor-partner interdependence mediation model. Res Nurs Health. 2018;41(2):156-165. https://doi.org/10.1002/nur.21860
S24. Gunnell DJ, Ewings P. Infertility prevalence, needs assessment and purchasing. J Public Health Med. 1994;16(1):29-35. https://doi.org/10.1093/oxfordjournals.pubmed.a042931
S25. Ried K, Alfred A. Quality of life, coping strategies and support needs of women seeking Traditional Chinese Medicine for infertility and viable pregnancy in Australia: a mixed methods approach. BMC Womens Health. 2013;13:17. https://doi.org/10.1186/1472-6874-13-17
S26. van Empel IW, Nelen WL, Tepe ET, van Laarhoven EA, Verhaak CM, Kremer JA. Weaknesses, strengths and needs in fertility care according to patients. Hum Reprod. 2010;25(1):142-149. https://doi.org/10.1093/humrep/dep362
S27. Mosalanejad L, Parandavar N, Gholami M, Abdollahifard S. Increasing and decreasing factors of hope in infertile women with failure in infertility treatment: a phenomenology study. Iran J Reprod Med. 2014;12(2):117-124.
S28. Deshpande NA, Braun IM, Meyer FL. Impact of fertility preservation counseling and treatment on psychological outcomes among women with cancer: a systematic review. Cancer. 2015;121(22):3938-3947. https://doi.org/10.1002/cncr.29637
S29. Letourneau JM, Ebbel EE, Katz PP, Katz A, Ai WZ, Chien AJ, et al. Pretreatment fertility counseling and fertility preservation improve quality of life in reproductive age women with cancer. Cancer. 2012;118(6):1710-1717. https://doi.org/10.1002/cncr.26459
S30. Bokaie M, Simbar M, Yassini Ardekani SM. Sexual behavior of infertile women: a qualitative study. Iran J Reprod Med. 2015;13(10):645-656.
S31. Kahlor L, Mackert M. Perceptions of infertility information and support sources among female patients who access the internet. Fertil Steril. 2009;91(1):83-90. https://doi.org/10.1016/j.fertnstert.2007.11.005
S32. Dyer SJ, Abrahams N, Hoffman M, van der Spuy ZM. Infertility in South Africa: women's reproductive health knowledge and treatment-seeking behaviour for involuntary childlessness. Hum Reprod. 2002;17(6):1657-1662. https://doi.org/10.1093/humrep/17.6.1657
S33. Sexton MB, Byrd MR, O'Donohue WT, Jacobs NN. Web-based treatment for infertility-related psychological distress. Arch Womens Ment Health. 2010;13(4):347-358. https://doi.org/10.1007/s00737-009-0142-x
S34. Sherrod RA. Understanding the emotional aspects of infertility: implications for nursing practice. J Psychosoc Nurs Ment Health Serv. 2004;42(3):40-47.
S35. Blenner JL. Health care providers' treatment approaches to culturally diverse infertile clients. J Transcult Nurs. 1991;2(2):24-31. https://doi.org/10.1177/104365969100200204
S36. Collopy K. Individualized decision support for women undergoing infertility treatment: an internet mediated needs assessment. Paper presented at: The 18th International Nursing Research Congress Focusing on Evidence-Based Practice; 2007 Jul 13-14; Vienna, Austria. Vienna: Sigma Theta Tau International, 2007.
